# Massive Splenomegaly in an Adult With a Hemoglobin Fractionation Pattern Suggestive of Hereditary Persistence of Fetal Hemoglobin: A Case Report

**DOI:** 10.7759/cureus.116885

**Published:** 2026-09-24

**Authors:** Dupati Madhusudan Rao, Vahini Golla, Sakthivadivel Varatharajan, Swarnalatha Seelam, Prasanth Raju

**Affiliations:** 1 Internal Medicine, All India Institute of Medical Sciences, Bibinagar, Bibinagar, IND; 2 General Medicine, All India Institute of Medical Sciences, Bibinagar, Bibinagar, IND; 3 Internal Medicine, Jawaharlal Institute of Postgraduate Medical Education and Research, Puducherry, IND; 4 Radiodiagnosis, All India Institute of Medical Sciences, Bibinagar, Bibinagar, IND; 5 Clinical Immunology and Rheumatology, ESIC Medical College and Hospital, Sanathnagar, Hyderabad, IND

**Keywords:** fetal hemoglobin, hemoglobinopathy, hereditary persistence of fetal hemoglobin, high-performance liquid chromatography (hplc), hpfh, massive splenomegaly

## Abstract

Hereditary persistence of fetal hemoglobin (HPFH) is an inherited hemoglobin disorder characterized by continued fetal hemoglobin (HbF) production after infancy and is generally associated with a clinically benign phenotype. Significant splenic enlargement is unusual and should prompt evaluation for other hematological, infectious, vascular, and hepatic causes. We report a 42-year-old man who presented with left-sided abdominal pain and marked splenomegaly, with the spleen palpable approximately 10 cm below the left costal margin. His hemoglobin remained relatively preserved, ranging from 10.9 to 13.6 g/dL, with normal leukocyte counts and intermittent mild thrombocytopenia. The reticulocyte production index was 0.2%, and the overall evaluation did not support active hemolysis. Screening for autoimmune disease, viral infections, and common infectious causes was negative. Abdominal ultrasonography and contrast-enhanced computed tomography demonstrated massive splenomegaly, splenic vein dilatation, and secondary left-sided hydronephrosis from extrinsic compression, while portal venous Doppler showed no evidence of portal hypertension. Hemoglobin analysis by high-performance liquid chromatography revealed complete predominance of HbF (100%) with no detectable adult hemoglobin (HbA) or adult hemoglobin A2 (HbA2), a pattern consistent with an HPFH phenotype. Bone marrow biopsy demonstrated preserved trilineage hematopoiesis without evidence of a primary hematological malignancy. The patient was managed conservatively with supportive treatment and was advised hematology follow-up and family screening. This case is notable for the coexistence of massive splenomegaly and an HPFH phenotype in an adult with relatively preserved hemoglobin and no convincing evidence of hemolysis. It highlights the importance of hemoglobin fractionation in the evaluation of unexplained splenomegaly and emphasizes that an unusual hemoglobin profile should be interpreted alongside the clinical phenotype and appropriate exclusion of alternative causes. Recognition of this benign hemoglobin phenotype may help avoid unnecessary invasive interventions when other causes of splenomegaly have been adequately excluded.

## Introduction

Hereditary persistence of fetal hemoglobin (HPFH) is an inherited condition in which production of fetal hemoglobin (HbF) continues into adult life beyond the normal developmental transition from γ-globin to β-globin expression. HPFH is genetically heterogeneous and may result from large deletions within the β-globin gene cluster or from sequence alterations affecting the regulatory regions of the γ-globin genes [1,2]. In most individuals, HPFH has a clinically benign phenotype, with little or no anemia or hemolysis despite persistent elevation of HbF [1,3].

Markedly elevated HbF in adulthood is not specific to HPFH and may also be observed in δβ-thalassemia, β-thalassemia, and other inherited or acquired conditions [3,4]. HPFH and δβ-thalassemia may have overlapping hemoglobin fractionation patterns, and definitive distinction may require family studies and molecular genetic analysis [4,5]. Distinguishing these conditions requires correlation of the HbF level with hemoglobin concentration, red-cell indices, other hemoglobin fractions, peripheral blood findings, and the clinical context [4,6]. Hemoglobin fractionation using high-performance liquid chromatography or electrophoretic techniques is an important component of this evaluation, although molecular analysis may be necessary when definitive genetic classification is required [4,5]. In the present case, the finding of 100% HbF with absence of adult hemoglobin (HbA) and adult hemoglobin A2 (HbA2) was suggestive of an HPFH phenotype; however, molecular confirmation was not performed. Therefore, the diagnosis is described as suspected HPFH rather than genetically confirmed HPFH. Although HPFH is generally associated with a benign hematological phenotype [4,6], the occurrence of marked splenomegaly creates an important diagnostic challenge and warrants systematic evaluation for alternative causes.

Massive splenomegaly is an unusual presentation in an otherwise preserved HPFH phenotype and warrants systematic evaluation for alternative causes, including hemolytic disorders, thalassemia syndromes, hematological malignancies, chronic infections, and portal hypertension. A previously reported adult with HPFH also presented with massive splenomegaly, illustrating that substantial splenic enlargement can occasionally accompany an HPFH phenotype [6]. We report an adult male who presented with symptomatic massive splenomegaly and an unusual hemoglobin fractionation pattern showing 100% HbF with absent HbA and HbA2. The case highlights the diagnostic challenge of massive splenomegaly in the presence of an atypical hemoglobin profile and emphasizes the importance of systematic evaluation and hemoglobin fractionation in reaching the diagnosis.

## Case presentation

A 42-year-old man, a farmer by occupation, belonging to a lower-middle-class family, presented with a 10-day history of left-sided abdominal pain. The pain was insidious in onset, dragging in character, nonradiating, and aggravated by coughing and deep inspiration. It was relieved by rest and lying down. He also reported easy fatigability. There was no history of fever, night sweats, weight loss, anorexia, bleeding manifestations, jaundice, hematemesis, melena, dyspnea, or previous blood transfusions. He denied the use of herbal medications. There was no known family history of hemoglobinopathies, and the patient was born out of a non-consanguineous marriage.

On examination, the patient was conscious, oriented, and hemodynamically stable. His vital parameters were within normal limits except for mildly elevated blood pressure. Abdominal examination revealed massive splenomegaly, with the spleen palpable approximately 10 cm below the left costal margin. There was no clinically significant hepatomegaly, ascites, or peripheral lymphadenopathy. Cardiovascular, respiratory, and neurological examinations were unremarkable.

Laboratory evaluation showed a hemoglobin level of 12.4 g/dL, red blood cell (RBC) count of 5.22 × 10^6^/µL, mean corpuscular volume (MCV) of 76.9 fL, mean corpuscular hemoglobin (MCH) of 24.8 pg, mean corpuscular hemoglobin concentration (MCHC) of 32.2 g/dL, and red cell distribution width-coefficient of variation (RDW-CV) of 20.2%. Total bilirubin was 2.08 mg/dL, with direct and indirect bilirubin levels of 0.70 and 1.38 mg/dL, respectively. Serum lactate dehydrogenase (LDH) was mildly elevated at 295 U/L (reference range: 120-246 U/L). The reticulocyte count was 0.5%, with an absolute reticulocyte count of 0.026 million/µL and a reticulocyte production index of 0.2%, indicating an inadequate reticulocyte response. Direct and indirect Coombs tests were negative. Peripheral smear showed predominantly normocytic, normochromic RBCs with a few microcytes, elliptocytes, and hypochromic cells, without abnormal RBCs. Serum haptoglobin was not performed because it was not available in our laboratory. Biochemical evaluation showed indirect hyperbilirubinemia with transient transaminitis. The available evaluation did not demonstrate evidence of a significant ongoing hemolytic process; however, serum haptoglobin was not available, and complete exclusion of hemolysis was therefore limited. Serum renal function and electrolytes were within normal limits. Autoimmune screening with antinuclear antibody testing was negative. Serological testing for HIV, hepatitis B virus, and hepatitis C virus was negative. The infectious evaluation, including testing for malaria, dengue, scrub typhus, leptospirosis, and enteric fever, was negative. The principal laboratory and diagnostic findings are summarized in Table 1. Hemoglobin fractionation was subsequently performed because of the markedly elevated HbF level in the setting of splenomegaly and relatively preserved hemoglobin. Hemoglobin electrophoresis and high-performance liquid chromatography demonstrated 100% HbF with no detectable HbA or HbA2, a pattern consistent with an HPFH phenotype (Figure 1). The laboratory report recommended genetic analysis and screening of family members for confirmation. In view of the marked splenomegaly, bone marrow biopsy was performed to exclude an underlying primary hematological malignancy. The biopsy showed mildly hypercellular marrow with increased erythropoiesis and micronormoblastic maturation, with preserved myelopoiesis and megakaryopoiesis, and no evidence of hematological malignancy (Figures 2, 3).

**Table 1:** Key Laboratory and Diagnostic Findings Summary of the principal hematological, biochemical, infectious, imaging, hemoglobin fractionation, and bone marrow findings identified during evaluation of massive splenomegaly. RBC: red blood cell; WBC: white blood cell; RDW-CV: red cell distribution width-coefficient of variation; LDH: lactate dehydrogenase; RPI: reticulocyte production index; ANA: antinuclear antibody; HBV: hepatitis B virus; HCV: hepatitis C virus; HbF: fetal hemoglobin; HbA: adult hemoglobin; HbA2: adult hemoglobin A2; HPFH: hereditary persistence of fetal hemoglobin

Investigation	Patient Value/Finding	Reference Range/Normal Value
Hemoglobin	10.9-13.6 g/dL	12-15 g/dL
RBC count	5.22 × 10^6^/µL	3.8-4.8 × 10^6^/µL
Total leukocyte count	5.10-10.08 × 10^3^/µL	4-10 × 10^3^/µL
Platelet count	112-209 × 10^3^/µL	150-410 × 10^3^/µL
Mean corpuscular volume	76.9 fL	83-101 fL
Mean corpuscular hemoglobin	24.8 pg	27-32 pg
Mean corpuscular hemoglobin concentration	32.2 g/dL	31.5-34.5 g/dL
RDW-CV	20.2%	11.6-14%
Peripheral blood smear	Predominantly normocytic, normochromic red cells, with few microcytes, elliptocytes, and hypochromic cells; no abnormal RBCs. WBC and platelet counts within normal limits. Hemoparasites not seen.	-
Reticulocyte count	0.5%	-
Absolute reticulocyte count	0.026 million/µL	-
Reticulocyte production index	0.2%	-
Serum LDH	295 U/L	120-246 U/L
Total bilirubin	2.08 mg/dL	0.2-1.3 mg/dL
Direct bilirubin	0.70 mg/dL	0.0-0.3 mg/dL
Indirect bilirubin	1.38 mg/dL	0.0-1.1 mg/dL
Direct Coombs test	Negative	Negative
Indirect Coombs test	Negative	Negative
Serum haptoglobin	Not performed; unavailable in our laboratory.	-
Serum iron	131 µg/dL	49-181 µg/dL
Serum ferritin	322 ng/mL	17.9-464 ng/mL
Total iron-binding capacity	220 µg/dL	261-462 µg/dL
Serum folate	1.44 ng/mL	2.76-20 ng/mL
Vitamin B12	338 pg/mL	239-931 pg/mL
Urea	Within normal limits	15-36 mg/dL
Creatinine	Within normal limits	0.52-1.04 mg/dL
Sodium	Within normal limits	137-145 mmol/L
Potassium	Within normal limits	3.5-5.1 mmol/L
Chloride	Within normal limits	98-107 mmol/L
ANA by immunofluorescence	Negative	Negative
HIV serology	Negative	Negative
HBV serology	Negative	Negative
HCV serology	Negative	Negative
Infectious workup	Malaria, dengue, scrub typhus, leptospirosis, and enteric fever negative	Negative
Portal vein Doppler	No evidence of portal hypertension.	No evidence of portal hypertension
Abdominal ultrasonography	Massive splenomegaly with mild hepatomegaly and left-sided hydronephrosis	-
Contrast-enhanced CT abdomen	Spleen 19.2 cm; liver 16 cm; prominent splenic vein measuring approximately 12 mm; mild left hydronephrosis with proximal ureteric narrowing, likely due to extrinsic compression by the enlarged spleen.	-
Hemoglobin fractionation	HbF 100%; HbA and HbA2 not detected.	HbF <1% in adults; HbA present; HbA2 2-3.5%
Bone marrow biopsy	Mildly hypercellular marrow with increased erythropoiesis and micronormoblastic maturation; preserved myelopoiesis and megakaryopoiesis.	-

**Figure 1:**
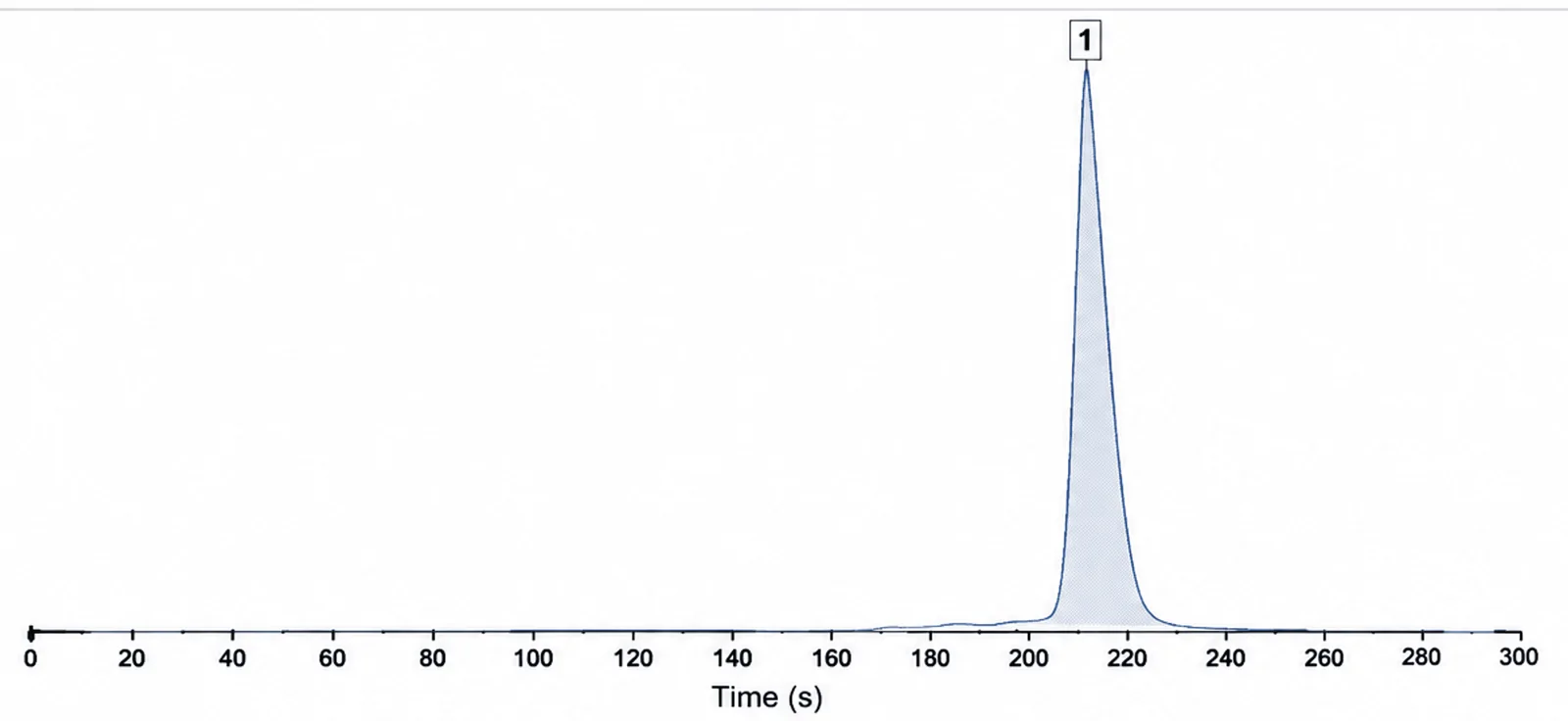
Hemoglobin Electrophoresis Demonstrating 100% HbF Hemoglobin electrophoresis showing a predominant HbF fraction of 100%, with the absence of HbA and HbA2. The reference range for HbF is <1%, and for HbA2 is 2.1-3.5%. HbF: fetal hemoglobin; HbA: adult hemoglobin; HbA2: adult hemoglobin A2

**Figure 2:**
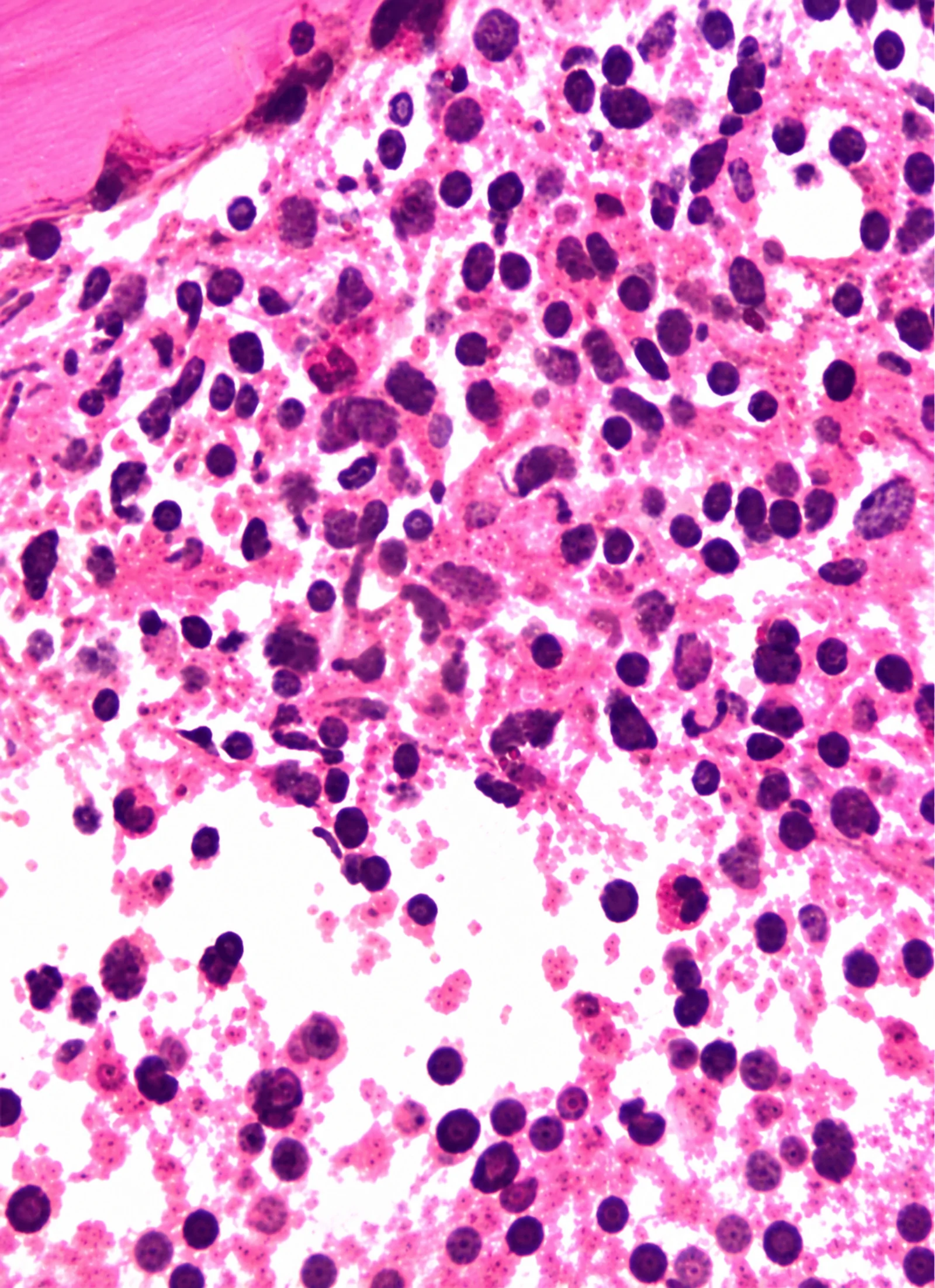
Bone Marrow Biopsy Demonstrating Trilineage Hematopoiesis With Erythroid Hyperplasia High-power view (400×, H&E) demonstrating increased erythropoiesis with micronormoblastic maturation, with preserved myeloid and megakaryocytic lineages.

**Figure 3:**
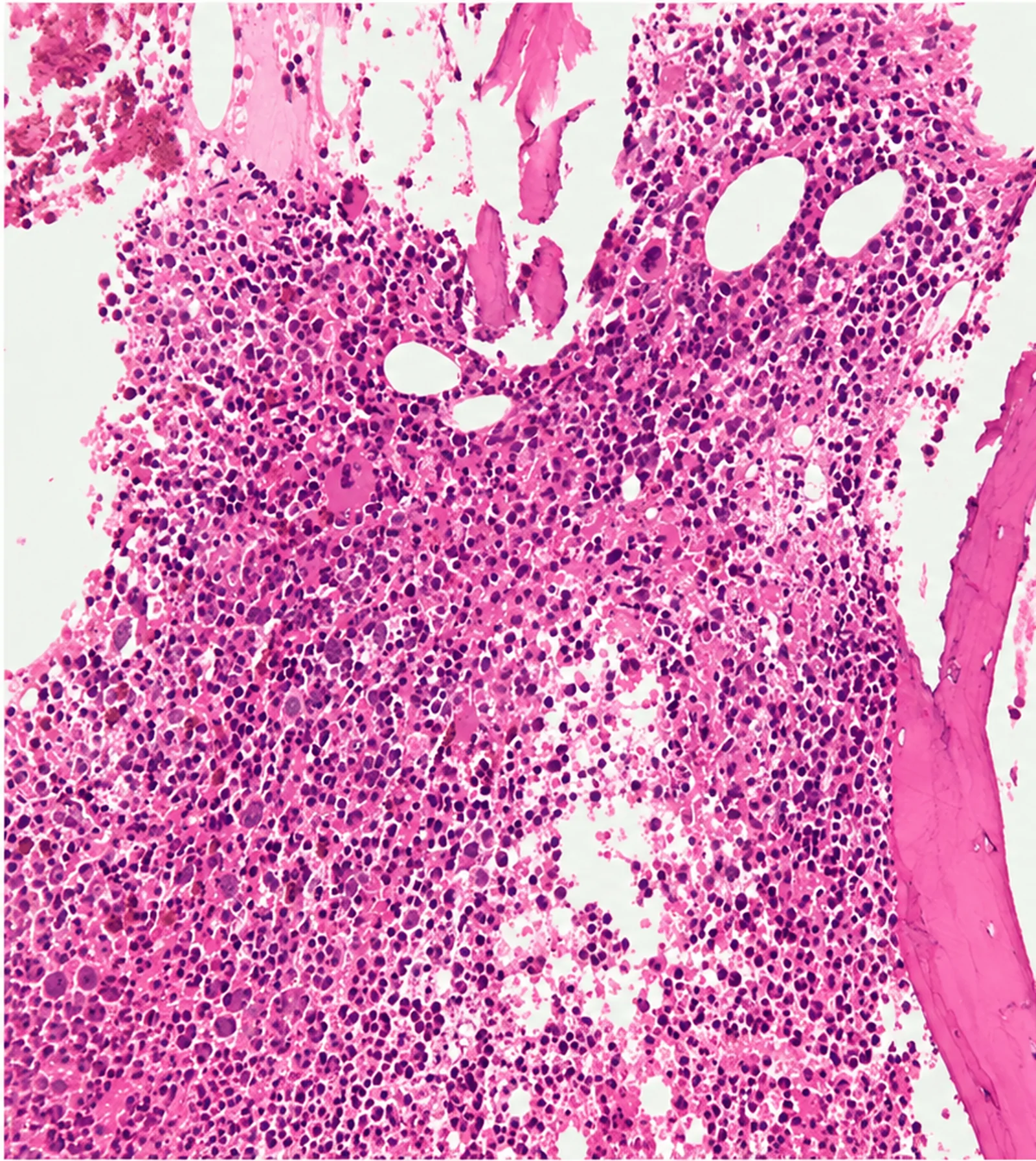
Bone Marrow Biopsy Showing Preserved Trilineage Hematopoiesis Low-power view (100×, H&E) showing mildly hypercellular marrow with preserved trilineage hematopoiesis.

Abdominal ultrasonography demonstrated massive splenomegaly with mild hepatomegaly and secondary left-sided hydronephrosis due to extrinsic compression. Portal venous Doppler showed no evidence of portal hypertension. Contrast-enhanced computed tomography of the abdomen demonstrated marked splenomegaly measuring 19.2 cm and mild hepatomegaly with the liver measuring 16 cm. The splenic vein was prominent, measuring approximately 12 mm, without evidence of thrombosis. Mild left hydronephrosis with sudden narrowing of the proximal ureter was noted, likely secondary to extrinsic compression by the enlarged spleen (Figures 4A, 4B).

**Figure 4:**
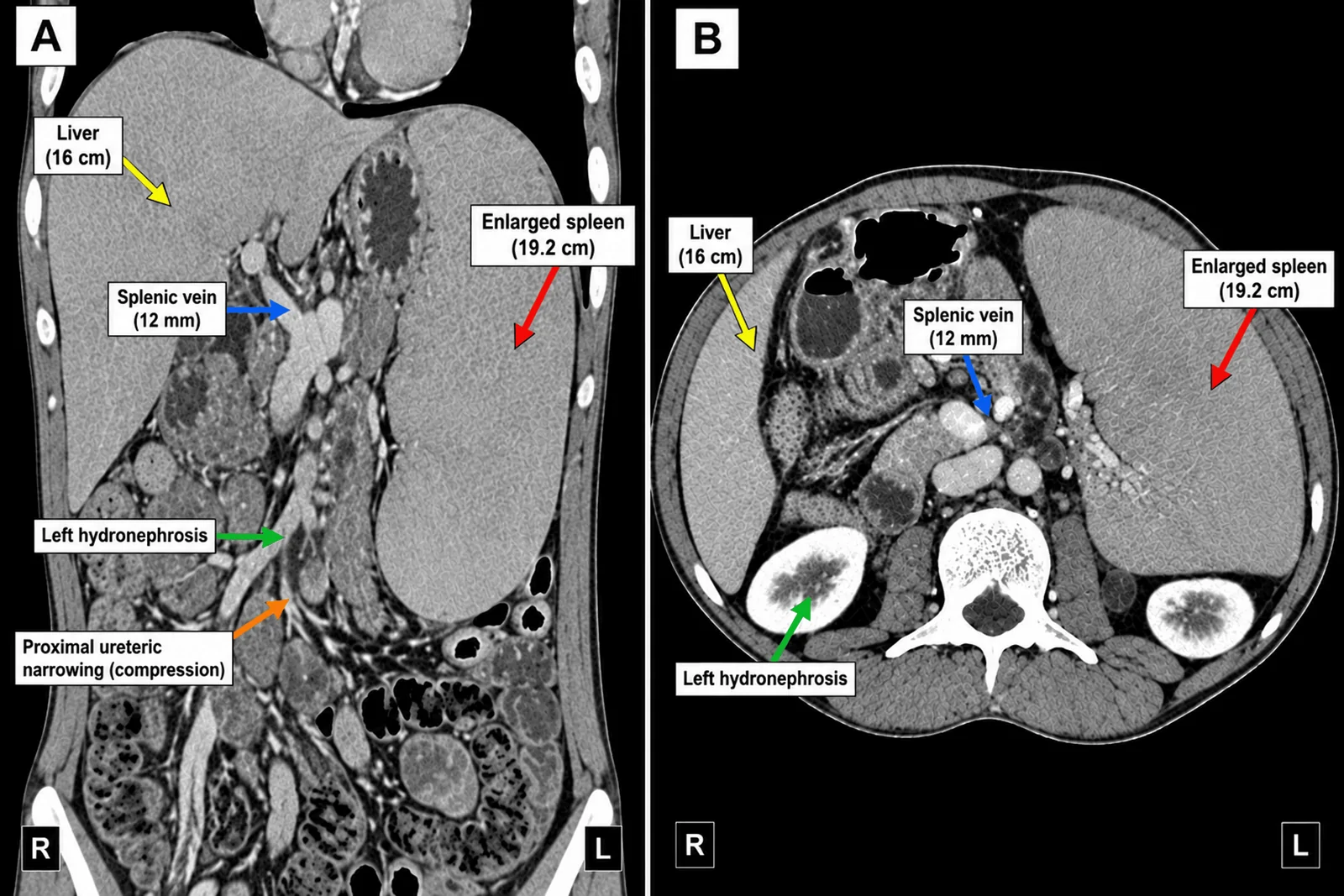
Contrast-Enhanced Computed Tomography of the Abdomen Showing Marked Splenomegaly (A) Coronal section demonstrating a markedly enlarged spleen with displacement and compression of adjacent abdominal structures. (B) Axial section demonstrating marked splenic enlargement with prominent splenic vasculature and associated mass effect. Mild left hydronephrosis with proximal ureteric narrowing, likely secondary to extrinsic compression by the enlarged spleen, is also noted.

Hemoglobin fractionation was subsequently performed because of the markedly elevated HbF level in the setting of splenomegaly and relatively preserved hemoglobin. Hemoglobin electrophoresis and high-performance liquid chromatography demonstrated 100% HbF with no detectable HbA or HbA2, a pattern consistent with an HPFH phenotype (Figure 1).

The laboratory report recommended genetic analysis and screening of family members for confirmation. In view of the marked splenomegaly, bone marrow biopsy was performed to exclude an underlying primary hematological malignancy. The biopsy showed mildly hypercellular marrow with increased erythropoiesis and micronormoblastic maturation, with preserved myelopoiesis and megakaryopoiesis and no evidence of hematological malignancy (Figures 2-3).

The differential diagnosis of massive splenomegaly was considered systematically. Hemolytic anemia was considered because of the indirect hyperbilirubinemia but was not supported by the low reticulocyte production index, relatively preserved hemoglobin, and negative Coombs testing. Thalassemia syndromes and other hemoglobinopathies were considered because of the markedly elevated HbF. Chronic infection, portal hypertension, autoimmune disease, and primary hematological malignancy were also evaluated and were not supported by the clinical, laboratory, imaging, and bone marrow findings. The major differential diagnoses and findings in this patient are summarized in Table 2.

**Table 2:** Differential Diagnosis and Evaluation of Massive Splenomegaly Major differential diagnoses considered during evaluation of massive splenomegaly and the findings that supported or argued against each diagnosis. HPFH: hereditary persistence of fetal hemoglobin; HbF: fetal hemoglobin; HbA: adult hemoglobin; HbA2: adult hemoglobin A2; RPI: reticulocyte production index; ANA: antinuclear antibody

Differential Diagnosis	Expected Clinical or Laboratory Findings	Findings in This Patient	Assessment
Hemolytic anemia	Anemia, reticulocytosis, indirect hyperbilirubinemia, and evidence of increased red-cell destruction [1]	Hemoglobin relatively preserved; RPI 0.2%; direct and indirect Coombs tests negative	Significant hemolysis not demonstrated
β-Thalassemia major/intermedia	Significant anemia, microcytosis, and markedly elevated HbF, with the hemoglobin fractionation pattern varying according to genotype [2]	Hemoglobin relatively preserved; HbF 100%; HbA2 not detected	Phenotype not typical
δβ-Thalassemia	Elevated HbF with variable microcytosis and anemia [3]	HbF 100% with relatively preserved hemoglobin	Less consistent with the observed phenotype
Myeloproliferative neoplasm or other primary hematological malignancy	Abnormal blood counts, constitutional symptoms, splenomegaly, or abnormal bone marrow findings [4]	No persistent leukocytosis; bone marrow showed trilineage hematopoiesis without malignancy	No evidence identified
Chronic infection	Fever, constitutional symptoms, relevant exposure history, or positive microbiological testing	No relevant systemic symptoms; infectious workup negative	Common infectious causes excluded
Portal hypertension	Ascites, varices, abnormal portal venous flow, or evidence of chronic liver disease	Portal vein Doppler showed no portal hypertension; no ascites or variceal bleeding	Portal hypertension not demonstrated
Autoimmune disease	Systemic features and supportive autoimmune serology	ANA negative; no supporting systemic features	Not supported
HPFH phenotype	Markedly elevated HbF with relatively preserved hemoglobin and a generally benign phenotype [5,6]	HbF 100%; HbA and HbA2 not detected; relatively preserved hemoglobin; no significant hemolysis	Most consistent with the observed hemoglobin phenotype

The patient was managed conservatively with analgesics, folate and vitamin B12 supplementation, low-dose aspirin, and supportive care. He remained hemodynamically stable and was discharged with advice for outpatient follow-up, review of the bone marrow findings, and family screening with hemoglobin fractionation.

## Discussion

HPFH is a genetically heterogeneous condition characterized by continued expression of γ-globin and persistence of HbF beyond infancy. Genetic mechanisms include large deletions involving the β-globin gene cluster and non-deletional variants affecting regulatory regions involved in γ-globin expression. The clinical consequences depend on the underlying molecular abnormality, but an isolated HPFH phenotype is generally associated with a mild phenotype because persistent HbF production can maintain effective oxygen carriage without producing substantial red-cell destruction [1,2]. The present case is unusual because the patient had marked splenomegaly despite relatively preserved hemoglobin and no convincing evidence of ongoing hemolysis.

The most striking laboratory finding was the presence of 100% HbF with absence of detectable HbA and HbA2. Such a pattern indicates profound persistence of HbF production and is compatible with an HPFH phenotype. However, hemoglobin fractionation alone cannot establish the precise molecular subtype of HPFH. Other inherited disorders characterized by markedly increased HbF may produce overlapping patterns, and definitive distinction may require correlation with red-cell indices, clinical phenotype, family studies, HbF distribution, and molecular genetic analysis [3-5]. In this patient, the relatively preserved hemoglobin concentration, low reticulocyte production index, absence of significant hemolytic features, and lack of a characteristic severe thalassemic phenotype favored an HPFH phenotype. Nevertheless, genetic testing was not performed, and therefore the exact molecular subtype cannot be established from the available data.

The second major diagnostic challenge was the presence of massive splenomegaly. HPFH is generally clinically silent, and substantial splenic enlargement is not considered a characteristic manifestation of uncomplicated disease. Consequently, the finding of massive splenomegaly required evaluation for other causes before attributing it to the hemoglobin phenotype. The differential diagnosis included hemolytic anemia, thalassemia syndromes, chronic infections, portal hypertension, autoimmune disease, and primary hematological malignancy. The patient's hemoglobin remained relatively preserved, while the reticulocyte production index was only 0.2%. Although indirect hyperbilirubinemia was present, the low reticulocyte response and negative direct and indirect Coombs tests did not support clinically significant ongoing hemolysis. The absence of persistent leukocytosis and the bone marrow findings further argued against a primary hematological malignancy.

The imaging findings were also important in defining the clinical significance of the splenomegaly. Ultrasonography and contrast-enhanced computed tomography confirmed marked splenic enlargement, while the latter demonstrated a prominent splenic vein and left-sided hydronephrosis with proximal ureteric narrowing related to extrinsic compression by the enlarged spleen. Portal venous Doppler showed no evidence of portal hypertension. Thus, the splenomegaly was not readily explained by portal hypertension, and its mechanical effect provided a plausible explanation for the patient's abdominal symptoms. The systematic exclusion of alternative causes was therefore central to the interpretation of the case.

The mechanism underlying the marked splenomegaly in this patient remains uncertain. Mild thrombocytopenia in the setting of marked splenomegaly raises the possibility of a hypersplenic component, although portal hypertension was not demonstrated. Extramedullary hematopoiesis is another theoretical mechanism in disorders associated with ineffective erythropoiesis; however, there was no direct radiological or pathological evidence of extramedullary hematopoiesis in this patient. Therefore, these mechanisms should be regarded as possible explanations rather than established causes of the splenomegaly.

The bone marrow examination had an additional diagnostic role because massive splenomegaly in an adult can be a manifestation of myeloproliferative or other hematological disorders. In this patient, the marrow was mildly hypercellular with increased erythropoiesis, while myelopoiesis and megakaryopoiesis were preserved. No evidence of hematological malignancy was identified. Together with the normal total leukocyte count and relatively preserved hemoglobin, these findings supported a nonmalignant explanation for the hematological abnormalities. The intermittent mild thrombocytopenia could potentially reflect splenic sequestration, although the available data do not establish hypersplenism as the cause.

The management approach was consequently conservative. Isolated HPFH generally does not require disease-specific treatment when it is clinically benign [1,2]. In this patient, treatment was directed toward symptomatic and nutritional support, with analgesics, folate, vitamin B12, low-dose aspirin, and supportive care. He remained hemodynamically stable and was discharged with follow-up advice. Splenectomy was not pursued because there was no demonstrated indication for surgical intervention, and the underlying mechanism of the splenomegaly remained uncertain. Family screening with hemoglobin fractionation was advised because HPFH is inherited and identification of a similar hemoglobin phenotype in relatives could provide additional clinical and genetic information.

An important limitation of this case is the absence of molecular confirmation. The hemoglobin fractionation pattern strongly supported an HPFH phenotype, but genetic analysis would have been valuable for determining whether the patient had a deletional or non-deletional form and for differentiating HPFH from other inherited causes of markedly elevated HbF. In addition, HbF distribution among individual erythrocytes was not assessed. These investigations could have strengthened the diagnostic classification and provided a clearer explanation of the patient's unusual phenotype.

This case highlights the importance of interpreting an extreme HbF elevation together with the complete clinical and hematological picture. In an adult with massive splenomegaly, an unusual hemoglobin profile should not immediately be assumed to explain the splenic enlargement. A structured evaluation for hemolysis, thalassemia syndromes, infection, portal hypertension, autoimmune disease, and hematological malignancy remains essential. In this patient, hemoglobin fractionation provided the key clue to an HPFH phenotype, while the broader investigation helped exclude several alternative causes of massive splenomegaly. The unusual coexistence of these findings emphasizes the value of comprehensive diagnostic evaluation and cautious interpretation when an uncommon hemoglobin phenotype is encountered.

## Conclusions

HPFH is generally a clinically benign inherited hemoglobin disorder, but its recognition can become challenging when it is encountered in a patient with massive splenomegaly. In this case, the combination of relatively preserved hemoglobin, absence of convincing ongoing hemolysis, extensive negative evaluation for infectious, portal hypertensive, autoimmune, and malignant causes, and hemoglobin fractionation showing 100% HbF with absent HbA and HbA2 supported an HPFH phenotype.

This case highlights the diagnostic challenge of evaluating marked splenomegaly in an adult with a hemoglobin fractionation pattern suggestive of an HPFH phenotype. The patient demonstrated 100% HbF with absent HbA and HbA2, relatively preserved hemoglobin levels, and no convincing evidence of a major ongoing hemolytic process or hematological malignancy. However, molecular genetic confirmation was not performed; therefore, a definitive diagnosis of HPFH or a specific genetic subtype cannot be established. The marked splenomegaly may be associated with the underlying hemoglobin phenotype, but a causal relationship cannot be established from this case. A systematic evaluation for infectious, hepatic, vascular, autoimmune, and hematological causes remains important, and management should be individualized according to symptoms, complications, and the underlying cause.
